# Comparative transcriptome analysis of the gills and hepatopancreas from *Macrobrachium rosenbergii* exposed to the heavy metal Cadmium (Cd^2+^)

**DOI:** 10.1038/s41598-021-95709-w

**Published:** 2021-08-09

**Authors:** Xue Liu, Hucheng Jiang, Baoqing Ye, Hongli Qian, Ziqi Guo, Haotian Bai, Jinhua Gong, Jianbin Feng, Keyi Ma

**Affiliations:** 1 Key Laboratory of Freshwater Aquatic Genetic Resources, Ministry of Agriculture (Shanghai Ocean University), College of Fisheries and Life Science, Shanghai Collaborative Innovation for Aquatic Animal Genetics and Breeding, Shanghai Ocean University, No. 999 Hucheng Huan Road, Pudong New Area, Shanghai, 201306 China; 2 Freshwater Fisheries Research Institute of Jiangsu Province, Nanjing, 210017 China; 3 Temasek Life Sciences Laboratory, Singapore, 117604 Republic of Singapore; 4 Dinghe Aquatic Science and Technology Development Co., LTD, Jiangsu, 225300 China

**Keywords:** Environmental impact, Biomarkers

## Abstract

Heavy metal Cadmium (Cd^2+^) pollution has become a severe environmental problem for aquatic organisms. In crustaceans, gills (Gi) and hepatopancreas (Hp) play a vital role in the toxicology. However, in *Macrobrachium rosenbergill*, there are few researches about gill and hepatopancreases responding to Cd^2+^ stress at a molecular level. In this study, transcriptomic analysis was applied to characterize gene expression profiles of gills and hepatopancreas of *M. rosenbergill* after Cd^2+^ exposure for 0 h, 3 h and 3 d. Six cDNA libraries (Gi 0 h, Gi 3 h, Gi 3 d, Hp 0 h, Hp 3 h, and Hp 3 d) were constructed and a total of 66,676 transcripts and 48,991 unigenes were annotated. Furthermore, differentially expressed genes (DEGs) were isolated by comparing the Cd^2+^ treated time-point libraries (3 h and 3 d group) with the control library (0 h group). The results showed that most of the DEGs were down-regulated after Cd^2+^ exposure and the number of DEGs among gill groups were significantly higher than those among hepatopancreas groups. GO functional and KEGG pathway analysis suggested many key DEGs in response to the Cd^2+^ stress, such as metallothionein and Hemocyanin. Additionally, a total of six DEGs were randomly selected to further identify their expressional profile by qPCR. The results indicated that these DEGs were involved in the response to Cd^2+^. This comparative transcriptome provides valuable molecular information on the mechanisms of responding to Cd^2+^ stress in *M. rosenbergii*, which lays the foundation for further understanding of heavy metal stress.

## Introduction

Over the past decades, aquatic heavy-metal pollution, such as copper (Cu^2+^), Zinc (Zn^2+^), and cadmium (Cd^2+^), has become one of the greatest concerns for aquatic environmental bio-monitoring worldwide^1^ due to its high toxicity, non-degradability, and subsequent bioaccumulation and biomagnification^2–5^. Cu^2+^ is an essential metal that participates in normal physiological process in crustaceans. But, the high concentrations of Cu^2+^ could induce the generation of reactive oxygen species (ROS), which lead to oxidative damage in many organisms^6,7^. Zn^2+^ is highly toxic for aquatic crustaceans and is a ubiquitous heavy metal in aquatic environment. High concentrations of Zn^2+^ inhibiting oxygen consumption had been reported in *Litopenaeus vannamei*^8^, *Litopenaeus schmitti*^9^, and *Farfantepenaeus paulensis*^10^. Among heavy metal pollutants, Cd^2+^ pollution ranks first in the world^11^, and it is toxic even at a very low concentration^12^. The toxic effects of cadmium have been well-documented in animals and humans. Cd^2+^ causes the accumulation of ROS which induce oxidization of biological macromolecules and results in various physiological damages to animal tissues and organs^13,14^. Furthermore, Cd^2+^ causes impairment of reproductive activity and disrupts endocrine function in fish^15^. Cd^2+^ induced cell apoptosis has been confirmed to be attributed to caspase-dependent and independent pathways of the mitochondria or endoplasmic reticulum (ER)^16,17^. Additionally, Cd^2+^ , a non-essential and potentially toxic metal, can be accumulated in humans via food chain^18^, which may result in morphological deformities, physiological dysfunctions and even death^19^. Previous researches showed that Cd^2+^ is known to accumulate in marine organisms and induced rapid genetic changes in many crustaceans, such as *Sinopotamon henanense*^19^ and *Eriocheir sinensis*^20^. Hence, it is essential to focus on the potential response mechanism caused by Cd^2+^ stress in crustaceans.

As an important respiratory organ, the gill (Gi) is involved in ion transport, acid–base balance and osmoregulation in crustaceans^21,22^. Due to the crustacean gills being exposed to the water in which they live, the gills play a vital role in the toxicological interactions, such as with heavy metals^23^. Furthermore, the hepatopancreas (Hp), a sensitive organ similar to the liver of higher organisms, is susceptible to be damaged by waterborne pollutants in crustaceans^24–26^. Therefore, gills and hepatopancreas are model organs for studying the response to heavy metal stress in crustaceans.

The giant freshwater prawn, *Macrobrachium rosenbergii*, is an important commercial prawn and widely cultured in China and other Pacific Rim countries^27^. As a freshwater cultured species, the prawn is susceptible to metal accumulation. Previous studies showed that structural changes of gills and hepatopancreas of *M. rosenbergii* could be caused by the Cu^2+^ accumulation, and the degree of damage observed was related to the elevated waterborne copper concentration^24^. Additionally, transcriptomic analysis of gills of *M. rosenbergii* showed that 19,417 and 8,989 differentially expressed genes (DEGs) were identified at 3 h and 48 h after Cu^2+^ exposure, respectively^28^, revealing that a large number of genes were involved in response to Cd^2+^ stress. Further research showed that the accumulation of Cd^2+^ also manifested histopathological changes in the gills and hepatopancreas of *M. rosenbergii* under Cd^2+^ exposure, and Cd^2+^ levels in tissues followed the order of: gills > hepatopancreas^29^. To date, however, limited researches were focused on the Cd^2+^-related stress response and regulatory gene in *M. rosenbergii*.

In this study, transcriptome sequencing of gills and hepatopancreas in *M. rosenbergii* was performed to analyze transcriptional responses under Cd^2+^ pollution. Many vital genes in response to the Cd^2+^ were identified. The study provided valuable and reliable data for aquaculture and environmental monitoring management, and elucidated the potential toxicological mechanism in *M. rosenbergii*.

## Results

### Transcriptome sequencing and functional gene annotation

**Figure 1:**
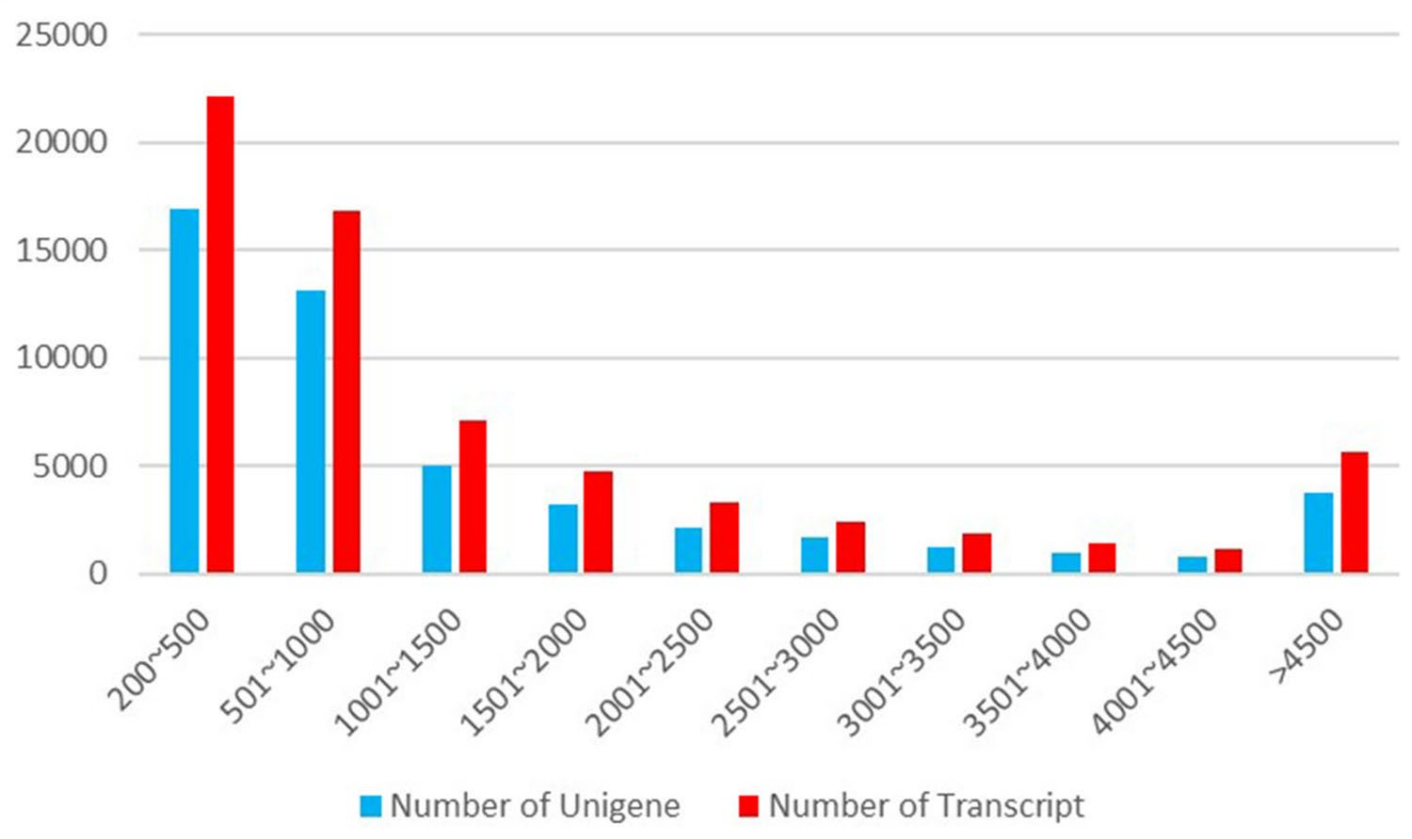
Length distribution of transcripts and unigenes. The X-axis indicates transcript and unigene size and the Y-axis indicates the number of transcript and unigene for each size.

Six cDNA libraries were constructed for Illumina sequencing and the sequencing generated 47,932,697, 45,863,583, 46,324,011, 44,082,407, 43,621,438, and 46,621,968 clean reads for Gi 0 h, Gi 3 h, Gi 3 d, Hp 0 h, Hp 3 h, and Hp 3 d, respectively (Table 1). The clean reads were assembled, and generated 66,676 transcripts, which were further clustered into 48,991 unigenes. The unigenes comprised of 74,217,621 bases, and the average length, largest length and smallest length were 1514.92 bp, 36,523 bp and 201 bp, respectively (Table 2). A length distribution of the total number of transcripts and unigenes is shown in Fig. 1. It is clearly displayed that there were 30,034 contigs (61.31%) ranging from 201 to 1000 bp, 15,172 contigs (30.97%) ranging from 1001 to 4500 bp, and 3785 contigs (7.73%) over 4,500 bp in length. Likewise, there were 38,933 transcripts (33.16%) ranging from 201 to 1000 bp, 22,109 transcripts (21.2%) ranging from 1,001 to 4,500 bp, and 5,634 transcripts (8.45%) over 4,500 bp in length. The transcript average coverage is 1599.88 bp, indicating that a high-quality transcriptome was assembled. Therefore, the assembled contigs in our study provide a useful resource for future research on *M. rosenbergii*.

**Table 1:** Summary of the sequencing data.

Sample	Raw reads	Clean reads	Error rate (%)	Q20 (%)	Q30 (%)	GC Content (%)
Gi0h_1	44,957,318	44,445,834	0.027	97.26	92.19	41.35
Gi0h_2	46,589,346	46,042,048	0.027	97.27	92.22	41.23
Gi0h_3	53,907,710	53,310,208	0.0267	97.39	92.48	41.29
Gi3d_1	41,266,892	40,659,896	0.027	97.24	92.22	41.9
Gi3d_2	49,137,222	48,468,382	0.0276	97.02	91.68	42.05
Gi3d_3	49,034,690	48,462,470	0.0269	97.33	92.37	42.07
Gi3h_1	47,657,670	47,101,762	0.0271	97.23	92.12	41.6
Gi3h_2	47,197,648	46,611,040	0.0273	97.14	91.95	41.66
Gi3h_3	45,785,340	45,259,232	0.0267	97.41	92.54	41.69
Hp0h_1	45,812,168	45,193,388	0.0268	97.35	92.43	43.52
Hp0h_2	45,040,828	44,414,802	0.0272	97.17	92.03	43.62
Hp0h_3	43,200,852	42,639,030	0.0268	97.35	92.46	43.65
Hp3d_1	45,569,698	44,981,812	0.0266	97.41	92.63	45.05
Hp3d_2	45,701,380	45,056,284	0.0266	97.39	92.58	45.2
Hp3d_3	41,305,288	40,826,218	0.0267	97.39	92.51	45.33
Hp3h_1	48,708,074	48,189,044	0.0262	97.59	93	44.55
Hp3h_2	46,612,070	46,115,142	0.0262	97.56	92.92	44.72
Hp3h_3	46,196,806	45,561,718	0.0266	97.38	92.64	44.51

Error rate (%): Percentage of the error bases; Q20: percentage of bases with a Phred value > 20; Q30: percentage of bases with a Phred value > 30. GC (%): percentage of bases G and C number in the total number of bases.

**Table 2:** The quality and length statistics of the transcripts and unigenes.

Type	Unigene	Transcript
Total number	48,991	66,676
Total base	74,217,621	106,673,706
Largest length (bp)	36,523	36,523
Smallest length (bp)	201	201
Average length (bp)	1514.92	1599.88
N50 length (bp)	3,093	3,223
E90N50 length (bp)	2,640	2,530

We annotated all unigenes with reference to six functional databases (Table 3) and found that 14,812 (30.23%), 11,869 (24.23%), 16,093 (32.85%), 18,536 (37.84%), 14,384 (29.36%) and 16,463 (33.6%) unigenes were mapped to GO, KEGG, COG, NR, Swiss-Prot, and Pfam, respectively.

**Table 3:** Annotation percentages of unigenes in different databases.

Annotated databases	All_Unigene number (percent)
GO	14,812 (0.3023)
KEGG	11,869 (0.2423)
COG	16,093 (0.3285)
NR	18,536 (0.3784)
Swiss-Prot	14,384 (0.2936)
Pfam	16,463 (0.336)
Total annotation	20,304 (0.4144)
Total	48,991 (1)

*GO* Gene Ontology, NR, *KEGG* Kyoto Encyclopedia of Genes and Genomes, *COG* clusters of orthologous groups of proteins, *NCBI* non-redundant protein sequences.

### Identification of differentially expressed genes (DEGs)

**Figure 2:**
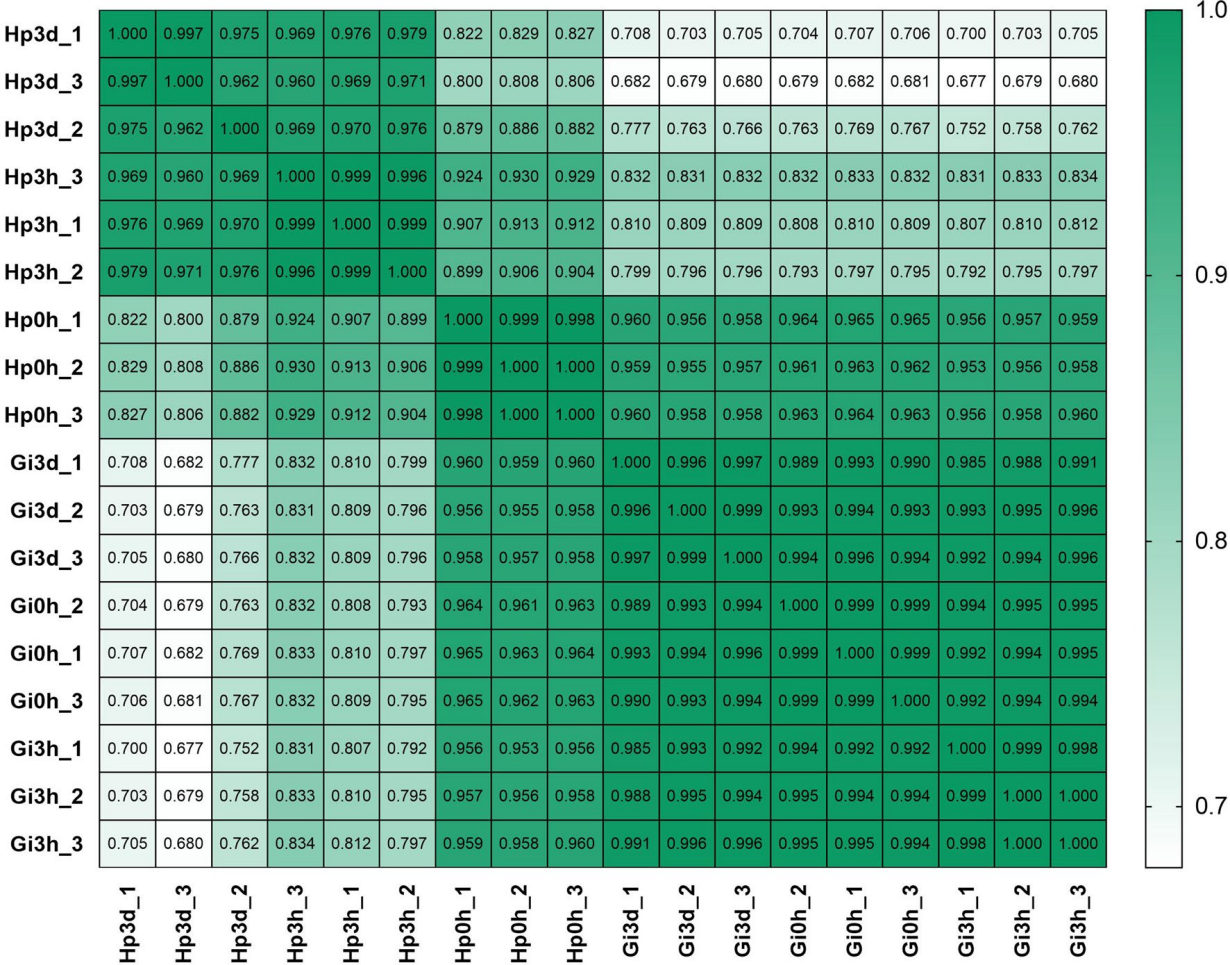
Pearson correlation analysis among the samples from different biological replicates. The abscissa and ordinate represent the sample name, and the correlation coefficient is represented by color; deeper color represents a stronger correlation.

**Figure 3:**
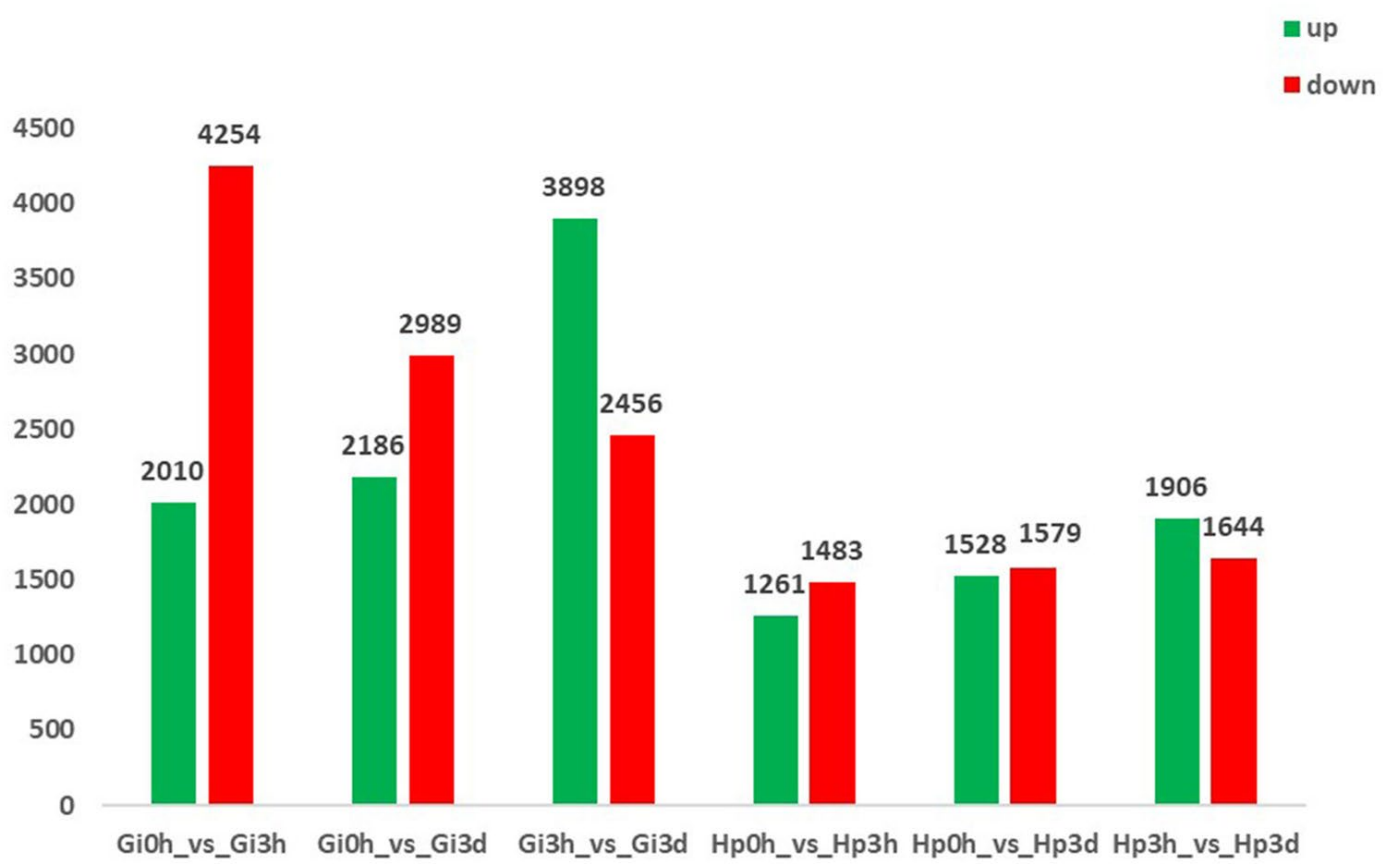
Differentially expressed genes (DEGs) in Cd^2+^ exposure and control groups. Y-axis represents the number of DEMs and X -axis represents different comparisons (Gi 0 h vs Gi 3 h, Gi 0 h vs Gi 3 d, Gi 3 h Vs Gi 3 d, Hp 0 h vs Hp 3 h, Hp 0 h vs Hp 3 d, and Hp 3 h vs Hp 3 d). Green dots represent up-regulated genes and red dots represent down-regulated genes. Gi, gill; *HP* hepatopancreas; 0 h, control; 3 h, Cd^2+^ treated 3 h; 3d, Cd^2+^ treated 3 days.

**Figure 4:**
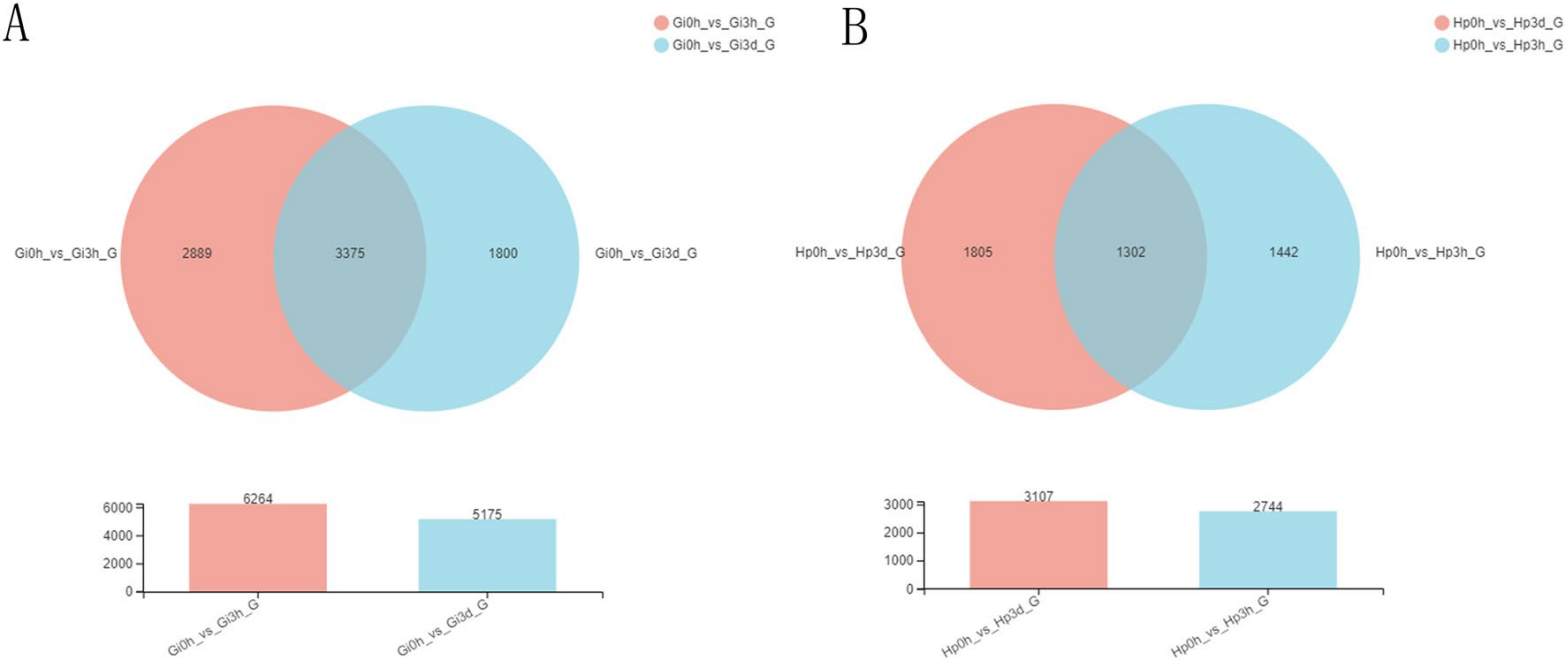
Venn diagram of differentially expressed genes (DEGs) in different comparisons. The overlaps represent the number of intersections of unigenes shared by the control group (0 h) and the two experimental groups (3 h and 3 d). Gi, gill; HP, hepatopancreas; 0 h, control; 3 h, Cd^2+^ treated 3 h; 3d, Cd^2+^ treated 3 days.

Pearson correlation analysis showed good correlation among different replicates of the same sample, whereas significant differences were observed between the gill and hepatopancreas groups (Fig. 2). To identify genes displaying significant changes in expression level in the face of Cd^2+^ stress, we analyzed the expression level of each unigene by TPM method and found many DEGs by comparing the Cd^2+^ treated time-points libraries (3 h and 3 d group) with the control library (0 h group) (Supplementary Table S1). Compared to gill control group (Gi 0 h), a total of 6264 (2,010 upregulated and 4254 downregulated) and 5175 (2,186 upregulated and 2989 downregulated) DEGs were identified in the Gi 3 h group and Gi 3 d group (Fig. 3), respectively. Long duration of Cd^2+^ exposure (Gi 3 d group) caused 4222 genes to be differentially expressed compared with short duration (Gi 3 h group) (Fig. 3). Furthermore, Venn analysis showed that 3375 genes were differentially expressed at both time-points, while 2889 DEGs were regulated just at Gi 3 h group and 1,800 genes were altered just at Gi 3 d group (Fig. 4). These time-specific genes might help to illustrate the stress response at different time-points. Similarly, many DEGs also existed between Hp 0 h and Hp 3 h, Hp 0 h and Hp 3 d, as well as Hp 3 h and Hp 3 h groups (Figs. 3, 4). Additionally, the number of DEGs among gill groups were significantly higher than those among hepatopancreas groups.

### GO functional annotation and KEGG enrichment analysis of DEGs

**Figure 5:**
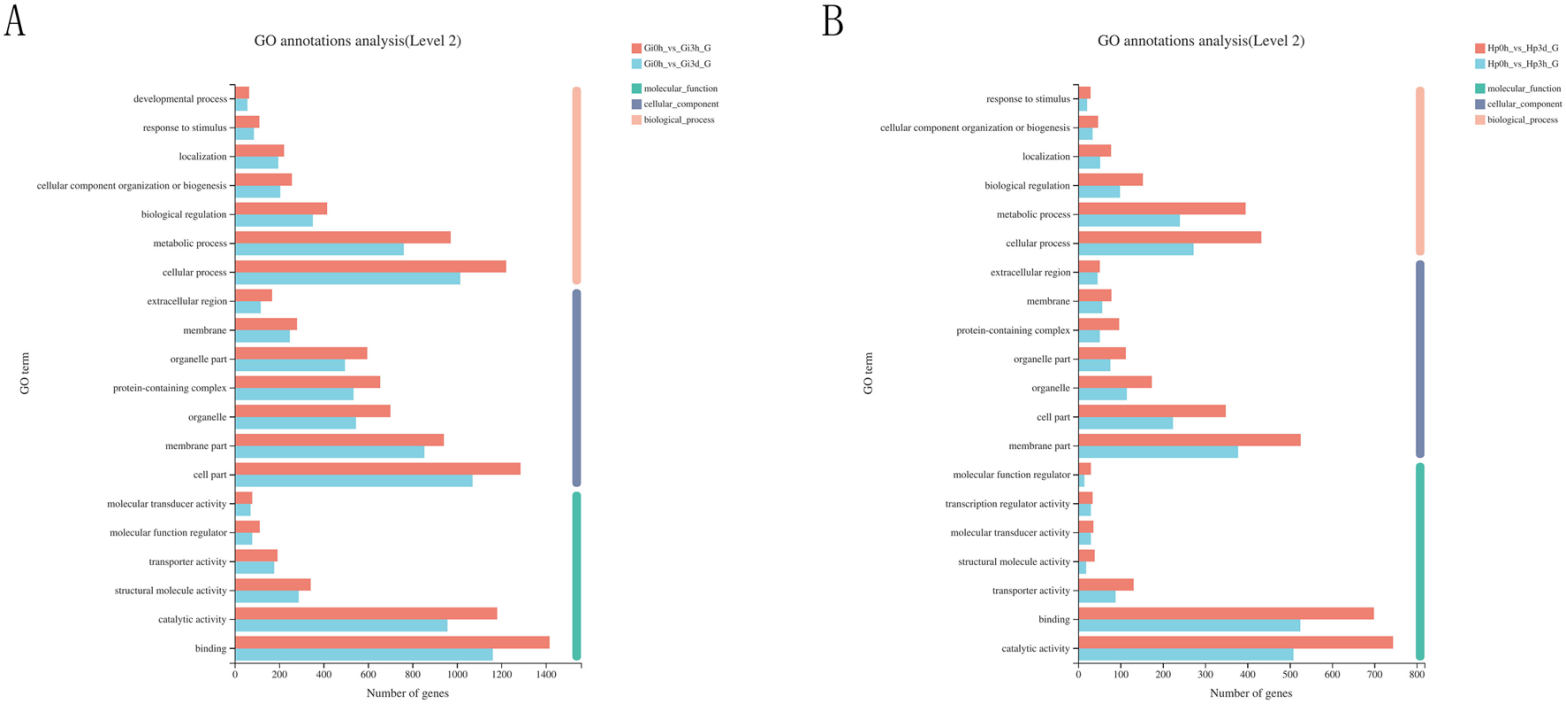
Gene ontology functional analyses of differentially expressed genes. (**a**) Gi 0 h vs Gi 3 h and Gi 0 h vs Gi 3 d. (**b**) Hp 0 h vs Hp 3 h and Hp 0 h vs Hp 3 d. Each annotated sequence is assigned at least one GO term from biological process, cellular component or molecular function. The abscissa represents the GO terms and the ordinate represents the number of genes. Gi, gill; HP, hepatopancreas; 0 h, control; 3 h, Cd^2+^ treated 3 h; 3d, Cd^2+^ treated 3 days.

**Figure 6:**
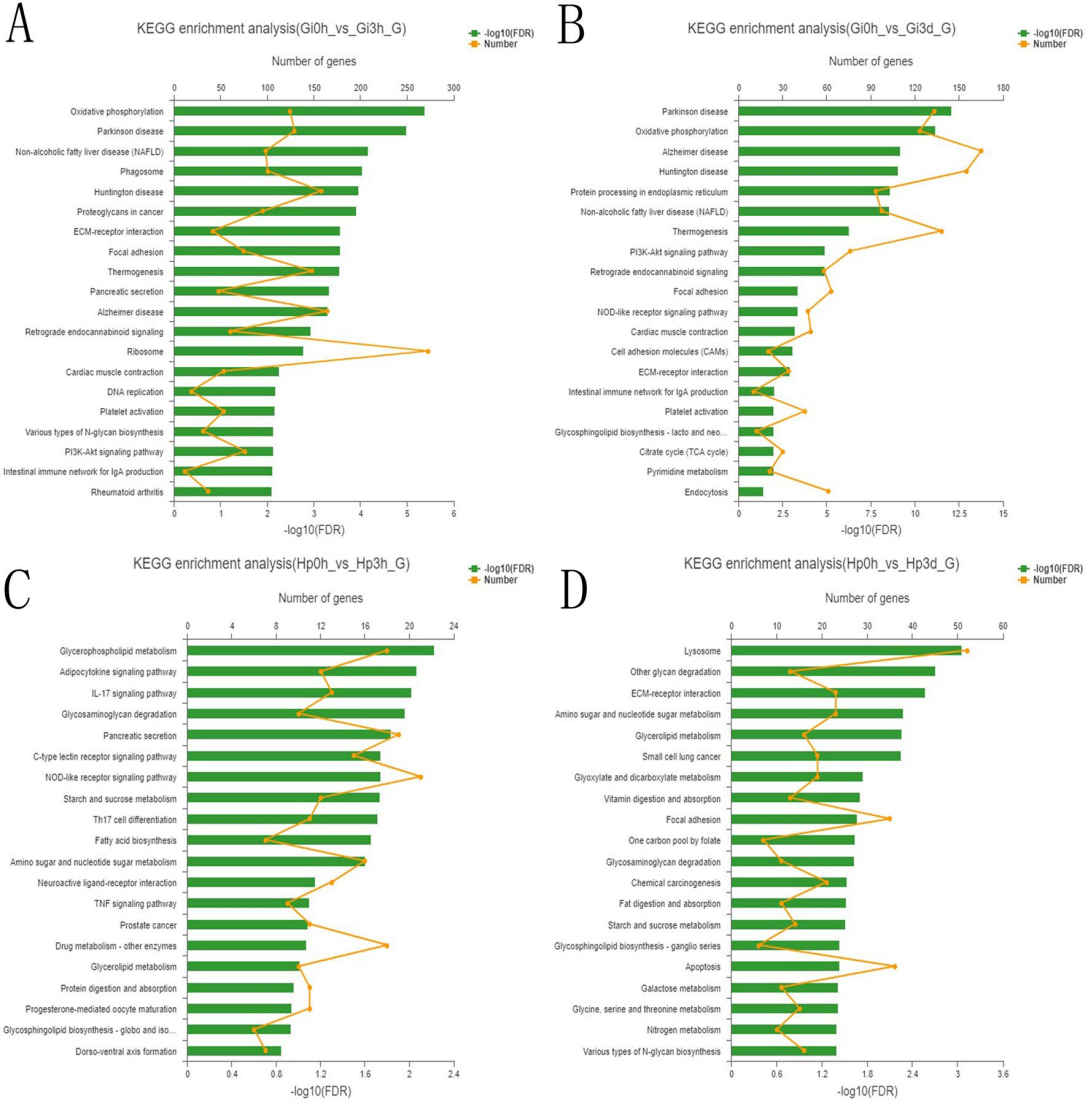
Kyoto Encyclopedia of Genes and Genomes functional analyses of differentially expressed genes between Gi 0 h and Gi 3 h (**a**), Gi 0 h and Gi 3 d (**b**), Hp 0 h and Hp 3 h (**c**), Hp 0 h and Hp 3 d (**d**). The ordinate shows the KEGG metabolic pathway and the broken line represents the number of genes. Gi, gill; HP, hepatopancreas; 0 h, control; 3 h, Cd^2+^ treated 3 h; 3d, Cd2 + treated 3 days. (The figures were created based on the KEGG pathway database www.kegg.jp/kegg/kegg1.html).

To better understand the biological functions and gene interaction of DEGs, all DEGs (Gi 0 h vs Gi 3 h, Gi 0 h vs Gi 3 d, Hp 0 h vs Hp 3 h and Hp 0 h vs Hp 3 d) were annotated in GO terms (Fig. 5). Among the categories of biological process, cellular component, and molecular function, the top 2 enriched GO terms for each category were “cellular process and metabolic process”, “membrane part and cell part”, and “catalytic activity and binding”, respectively. A total of 59 genes were annotated to “response to oxidative stress” GO terms (Supplementary Table S2), such as catalase, peroxidase and NADH dehydrogenase. Subsequently, KEGG pathway analysis was performed to identify the functions of DEGs and biological pathways involved in metal stress response. The top 20 significantly enriched KEGG pathways in each comparison are shown in Fig. 6. For the gill groups, 13 of those pathways (Intestinal immune network for IgA production, ECM-receptor interaction, Platelet activation, Cardiac muscle contraction, Retrograde endocannabinoid signaling, Focal adhesion, PI3K-Akt signaling pathway, Non-alcoholic fatty liver disease (NAFLD), Oxidative phosphorylation, Parkinson disease, Thermogenesis, Huntington disease, and Alzheimer disease) were enriched in both Gi 3 h and Gi 3 d compared with Gi 0 h group, whereas for the hepatopancreas groups, only 4 pathways (Glycosaminoglycan degradation, Starch and sucrose metabolism, Amino sugar and nucleotide sugar metabolism, and Glycerolipid metabolism) were enriched in both Hp 3 h and Hp 3 d compared with Hp 0 h group. “Ribosome (272 genes)”, “Alzheimer disease (165 genes)”, “NOD-like receptor signaling pathway (21 genes)”, and “lysosome (52 genes)” were significantly the most gene enriched pathways in Gi 0 h vs Gi 3 h, Gi 0 h vs Gi 3 d, Hp 0 h vs Hp 3 h, and Hp 0 h vs Hp 3 d comparisons, respectively. These enriched pathways may play important roles in metal stress response. GO functional and KEGG analyses of DEGS (Gi 3 h vs Gi 3 d and Hp 3 h and Hp 3 d) are shown in Supplementary Figure S1.

### Validation of DEGs by qPCR

**Figure 7:**
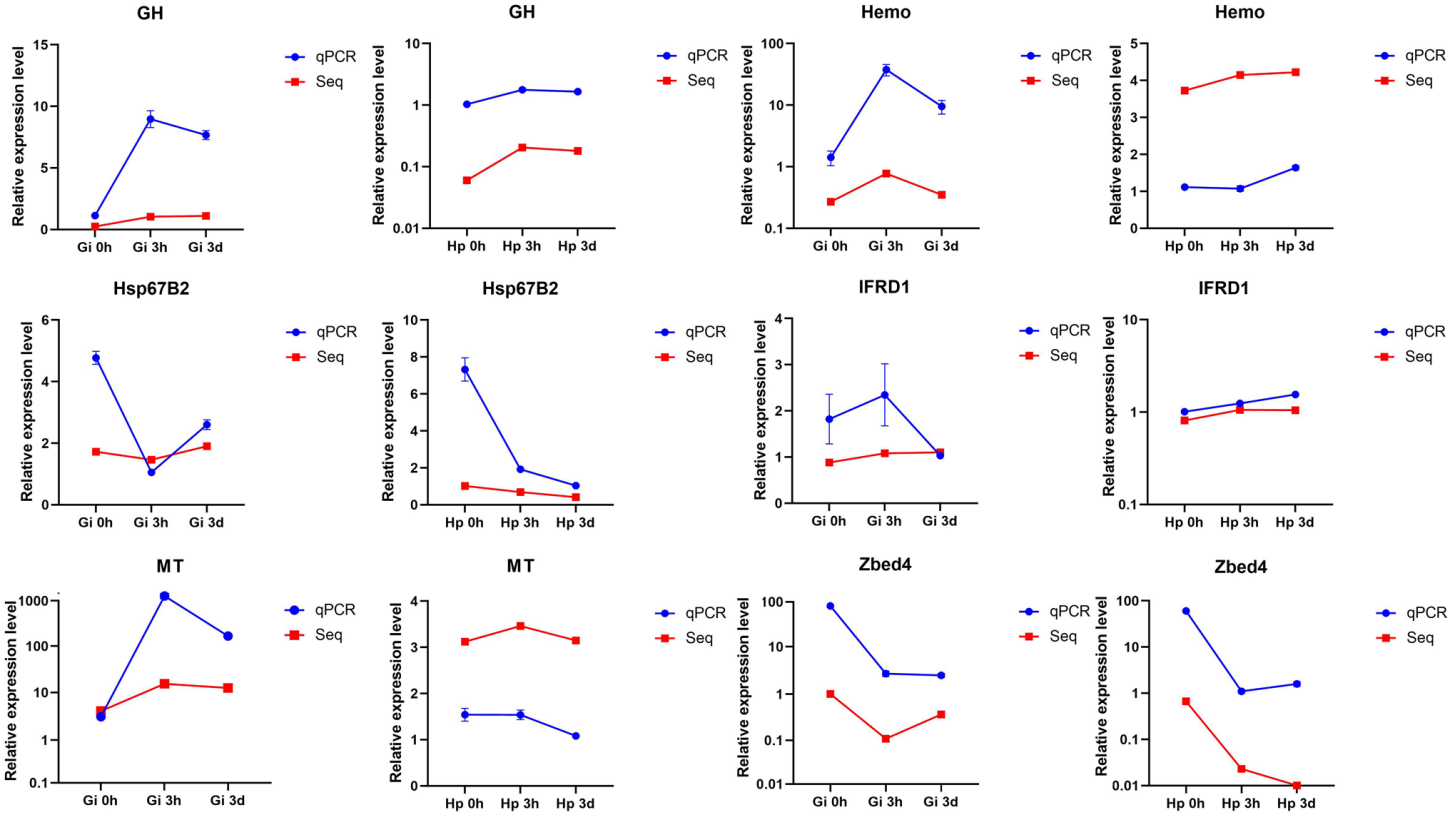
Validation of the relative expression level of six genes associated with Cd^2+^ stress using quantitative real time PCR. Blue bar represents the qPCR results, and red bar represents the RNA-Seq results. *MT* metallothionein, *Hemo* hemocyanin-like protein, *IFRD1* interferon-related developmental regulator 1, *Hsp67B2* heat shock protein 67B2-like, *Zbed4* zinc finger BED domain-containing protein 4-like, *GH* gamma-glutamyl hydrolase-like.

The general trend of six DEGs (MT, Hemo, IFRD1, Hsp 67B2, Zbed4, and GH) involved in metal transportation and stress response was in accordance with the results from RNA sequencing (Fig. 7). Obviously, the change range of expression level of many DEGs in gill treated groups is greater than those in hepatopancreas treated groups compared to control group. For example, the expression level of GH gene significantly increased by eight and seven times in gill at 3 h and 3 d group, respectively. In addition, the DEGs present various expression patterns in gill and hepatopancreas after Cd^2+^ exposure. For instance, the expression level of Hemo significantly increased in gill at 3 h and then decreased at 3d, but consistently increased in hepatopancreas under Cd^2+^ stress. In contrast, the expression level of Hsp67B2 gene significantly decreased in gill at 3 h and then increased at 3 d, but consistently increased in hepatopancreas under Cd^2+^ stress.

## Discussion

In crustaceans, the gill epithelium is generally regarded as a major organ of respiration and osmoregulation, and the first site to be exposed to environmental pollutants^30^. During waterborne exposure to heavy metals, gills act as a protective barrier between the internal and external environment^23^. Waterborne heavy metals are initially absorbed into epithelium cells of gill and transported into hemolymph, and finally infiltrated into internal organs^23^. Hepatopancreas is usually considered as a vital target organ for heavy metal toxicity and other environmental stresses in crustaceans and plays a major role in metal storage and in the detoxification process^31^. Additionally, crustaceans increase metabolic efficiency by promoting the digestive enzyme activities in hepatopancreas in response to heavy metal^32^. Therefore, the gill and hepatopancreas are considered as a good indicator of water quality, and a suitable model for studies of heavy metal pollution.

To better understand the molecular mechanisms of Cd^2+^ toxicity in *M. rosenbergii*., RNA-Seq was used to investigate gene expression differences of gill and hepatopancreas in response to Cd^2+^ exposure (0 h, 3 h, and 3 d). Six cDNA libraries were constructed and a total of 48,991 unigenes were functionally annotated. GO term enrichment and KEGG pathway enrichment were performed to find important genes and pathways during Cd^2+^ exposure in gill and hepatopancreas of *M. rosenbergii*. We analyzed DEGs by comparing the Cd^2+^ treated time-point libraries with the control library. The results showed that the number of down-regulated DEGs is larger than up-regulated DEGs (Fig. 3), indicating that gene expressions were mainly inhibited by Cd^2+^, which leads to impairments in *M. rosenbergii*. The results were similar with *Sinopotamon henanense* and *Danio rerio* under Cd^2+^ stress^19,33^. The number of DEGs among Gi groups were significantly higher than those among Hp groups (Fig. 3), suggesting that the gill has a stronger stress response than hepatopancreas in short time. Additionally, the number of DEGs in Gi groups decreased with the increment of exposure time, while in Hp groups, the number of DEGs increased with the increment of exposure time (Fig. 3). The above results might be attributed to the reason that the gill acts as the entry site and transient store organ of the heavy metal for a short period of exposure time, and Cd^2+^ is gradually transferred from the gills to hepatopancreas via the haemolymph with the prolongation of exposure time^34^.

Many genes related to oxidative stress were found in response to the Cd^2+^ stress (Supplementary Table S2), and present various expression patterns, as identified by qPCR (Fig. 7). For example, the expression level of metallothionein (MT) was significantly increased at 3 h, then decreased at 3d, which may be related to the accumulation of Cd^2+^. Many studies have shown that MT is critical to heavy metal detoxification^35,36^ in addition to storage of essential elements that are necessary for metalloenzymes^37,38^. Some studies have proven that the accumulation of heavy metal has significant time effects. For instance, in *Oncorhynchus mykiss*, Cu^2+^ uptake increased during the 1–2 h under radiolabelled copper exposure, and after 2 h, Cu^2+^ level significantly decreased in the gill^39^. A similar tendency was found in *Acrossocheilus fasciatus*^40^, in which the expression level of zinc-finger BED domain-containing protein (Zbed) was significantly decreased after exposure to Cd^2+^, which is also consistent with what has been observed in *Mytilus galloprovincialis* exposed to Cu^2+^^41^. In contrast, hemocyanin-like protein, a crucial immune protein in arthropods^42–44^, had significantly increased expression after exposure to Cd^2+^. Heavy metals are handled through separate metabolic pathways dependent on hemocyanin^45^. In addition, the expression level of heat shock proteins (Hsps), common stress-inducible proteins, has been known to increase under various stressors, such as oxidative stress, heavy metals, and viral infections^46–48^. For instance, Hsp70, Hsp40, and Hsp105, were significantly up-regulated in *Eubalaena glacialis* exposed to Cd^2+^. Interestingly, in *M. rosenbergii,* Hsp67B2 was consistently decreased in the hepatopancreas for three days under Cd^2+^ exposure, suggesting that Hsp67B2 may be suppressed by Cd^2+^ in this prawn*.* On the other hand, the expression level of IFRD1 was consistently increased in hepatopancreas under Cd^2+^ stress, which was consistent with the high upregulation of this gene in hepatopancreas of *M. rosenbergii* after virus infection^49^. IFRD1 protein has been proven to be involved in the regulation of inflammatory responses^50^, indicating that the increased expression of IFRD1 is intended to cure inflammation caused by Cd^2+^.

Nevertheless, further study is required to illustrate the regulatory mechanism of *M. rosenbergii* after exposure to Cd^2+^. The degree of histological damage of the gills and hepatopancreas under different concentrations and exposure days of Cd^2+^ is worth exploring in future research. Additionally, the effects of Cd^2+^ on the mitochondrion structure in the gill and on superoxide dismutase (SOD) activity still need to be investigated.

## Conclusion

In conclusion, we successfully constructed comparative gill and hepatopancreas transcriptome datasets in Cd^2+^ treated groups and control group of *M. rosenbergii*. Thereafter, 48,991 unigenes were functionally annotated and a series of DEGs were isolated after Cd^2+^ exposure. Based on GO functional and KEGG pathway analyses, many DEGs that are potentially relevant to immune responses, antioxidant, and detoxification were identified.

## Material and methods

### Collection and maintenance of prawns

A total of nine female and nine male *M. rosenbergii* (23 ± 2.5 g) individuals used in this experiment were collected from Dinghe Aquatic Science and Technology Development Co. LTD (Jiangsu, China) and transported back to our laboratory. The prawns were maintained at 26 ± 2 °C in a 50-L aerated aquarium for three days before treatment. All animals were handled in accordance with guidelines established by the Animal Experiments Ethics Committee of Shanghai Ocean University for the care and use of laboratory animals.

### Cadmium exposure experiment

Firstly, Cd^2+^ solution (50 mg/L) was prepared by dissolving 102 mg of CdCl_2_·2.5 H_2_O in 1 L deionized water. After temporary rearing, CdCl_2_ solution was added to the culture water and mixed immediately, so as to expose all the prawns to the Cd^2+^ (80 μg/L) based on the 96-h LC50 of Cd in *M. rosenbergii*^51^. Every day, the prawns were fed and the water was renewed by 50% to maintain water quality. Subsequently, the experimental prawns were anesthetized on ice and dissected. The gills (Gi) and hepatopancreas (Hp) were randomly sampled from six individuals (three males and three females) for each of the 3 time points: 0 h, 3 h and 3 d, after Cadmium exposure, and stored at − 80 °C immediately for the following RNA extraction.

### Library construction and gene function annotation

Total RNA was extracted from gills and hepatopancreas using Trizol reagent (Invitrogen, USA). The purity and amount of the 36 RNA samples were assessed by NanoDrop2000C, and RNA integrity was verified by agarose electrophoresis. The RNA integrity was assessed by Agilent 2100 (RIN number > 6.5). For each group, equal amount of RNA from the six individuals were pooled to make a sample for library construction. Three replicates were conducted for each library for statistics and comparison. Subsequently, the mRNA was purified and submitted to synthesize cDNA. Finally, the resulting six libraries (Gi 0 h, Gi 3 h, Gi 3 d, Hp 0 h, Hp 3 h, and Hp 3 d) were sequenced on Illumina Hiseq platform at Maiorbio company (Shanghai, China). Raw reads were trimmed by deleting adapter, ploy-N and low-quality reads. Then, the remaining clean reads were assembled into longer contigs using Trinity. The longest transcripts of each gene were defined as unigenes. Assembled unigenes were annotated by comparison to six databases, including NCBI non-redundant protein database (NR), Swiss-Prot, Pfam, Cluster of Orthologous Groups of proteins (COG), Gene Ontology (GO), and Kyoto Encyclopedia of Genes and Genomes (KEGG) (http://www.genome.jp/kegg/pathway.html)^52–54^. The RNA-seq data have been deposited in the NCBI database under the accession number PRJNA707962.

### Gene expression analysis

Gene expression values were calculated, using RSEM and measured as transcripts per kilobase per million mapped reads (TPM), for the six cDNA libraries. Correlation coefficients of samples were computed using Pearson correlation. DESeq2 (with parameters of: p-value < 0.05 and fold-change ≥ 2) was applied for analysis of differentially expressed genes (DEGs). The DEGs between the library pairs (Gi 0 h vs Gi 3 h, Gi 0 h vs Gi 3 d, Gi 3 h vs Gi 3 d Hp 0 h vs Hp 3 h, Hp 0 h vs Hp 3 d, and Hp 3 h vs Hp 3 d) were identified. Then GO and KEGG functional classification were performed to identify which DEGs were significantly enriched in GO terms and metabolic pathways.

### Validation of DEGs expression profiles using quantitative real-time RT-PCR (qPCR)

To validate the Illumina sequencing results, the six pooled RNA samples originally used for transcriptome sequencing were analyzed by qPCR. Six randomly selected genes: metallothionein (MT), hemocyanin-like protein (Hemo), interferon-related developmental regulator 1 (IFRD1), heat shock protein 67B2 (Hsp 67B2), zinc finger BED domain-containing protein 4 (Zbed4), and gamma-glutamyl hydrolase (GH), were amplified by specific primers (Table 4). QPCR mixture (20 μL) contained 10 μL of PCR Master with SYRB green, 1 μL Cd^2+^ cDNA template (10 ng/ul), 0.25 μL of each primer (10 uM), and 8.5 μL H_2_O. The primers of *β-actin* were used as the internal control. The relative quantification of the six genes was calculated by the 2-^△△^CT method^55^. Analysis of qPCR results was performed in GraphPad Prism 8. All data were presented as means ± SD.

**Table 4:** Real-time quantitative PCR primers used in this study.

Gene	Forward primer sequence (5′–3′)	Reverse primer sequence (5′–3′)
*β-actin*	CGACGGTCAGGTCATCACCA	ACGTCGCACTTCATGATGGA
MT	ACTCAGATTTTCTCAGCACCA	CTGAAAAACGGAACAACATGA
hemo	TTATGGTGCCCTCCACAACTT	TGAAGAATGCAGGATCACGAGT
IFRD1	TGCTCTTTGTTGCTTTCGGTC	CTCTCAATGGCTTCTGTCTCCTC
Hsp67B2	CTACGGGTCGAGGGAACTTGA	CGATTCCGCCCTTAGATTTTG
Zbed4	CTATGGCACTTAGATGGGGGA	GAAACAACACAGAAGGGCTCA
GH	TGATGGAAAAGCCTAAGCGAG	AGTCTATGTCAATTATGCCCG

*MT* metallothionein, *Hemo* hemocyanin-like protein, *IFRD1* interferon-related developmental regulator 1, *Hsp67B2* heat shock protein 67B2-like, *Zbed4* zinc finger BED domain-containing protein 4-like, *GH* gamma-glutamyl hydrolase-like.

### Approval statement

All experimental protocols were approved by the Key Laboratory of Freshwater Aquatic Genetic Resources, Ministry of Agriculture, Shanghai Ocean University in this paper.

### ARRIVE guidelines statement

This study was carried out in compliance with the ARRIVE guidelines.

## Supplementary Information

Supplementary Information 1.

Supplementary Information 2.

Supplementary Information 3.

Supplementary Information 4.
