# Supplementary material for: Comparative transcriptome analysis of the gills and hepatopancreas from Macrobrachium rosenbergii exposed to the heavy metal Cadmium (Cd2+)
Source: Sci Rep. 2021 Aug 9;11:16140. doi: 10.1038/s41598-021-95709-w (PMC8352946; doi:10.1038/s41598-021-95709-w)
Supplement: Supplementary file 1 — Supplementary Information 1. [file 41598_2021_95709_MOESM1_ESM.docx]

**Additional Information**

**Supplementary Figure S1** GO functional and KEGG analyses of DEGS (Gi 3 h vs Gi 3 d and Hp 3 h and Hp 3 d)

**Supplementary Table S1** Annotation details of all the DEGs by comparing the Cd2+ treated time-point libraries (3 h and 3 d group) with the control library (0 h group)

**Supplementary Table S2** Differentially expressed genes (DEGs) related to oxidative stress.
