# Supplementary figures and images for: Comparative transcriptome analysis of the gills and hepatopancreas from Macrobrachium rosenbergii exposed to the heavy metal Cadmium (Cd2+)
Source: Sci Rep. 2021 Aug 9;11:16140. doi: 10.1038/s41598-021-95709-w (PMC8352946; doi:10.1038/s41598-021-95709-w)

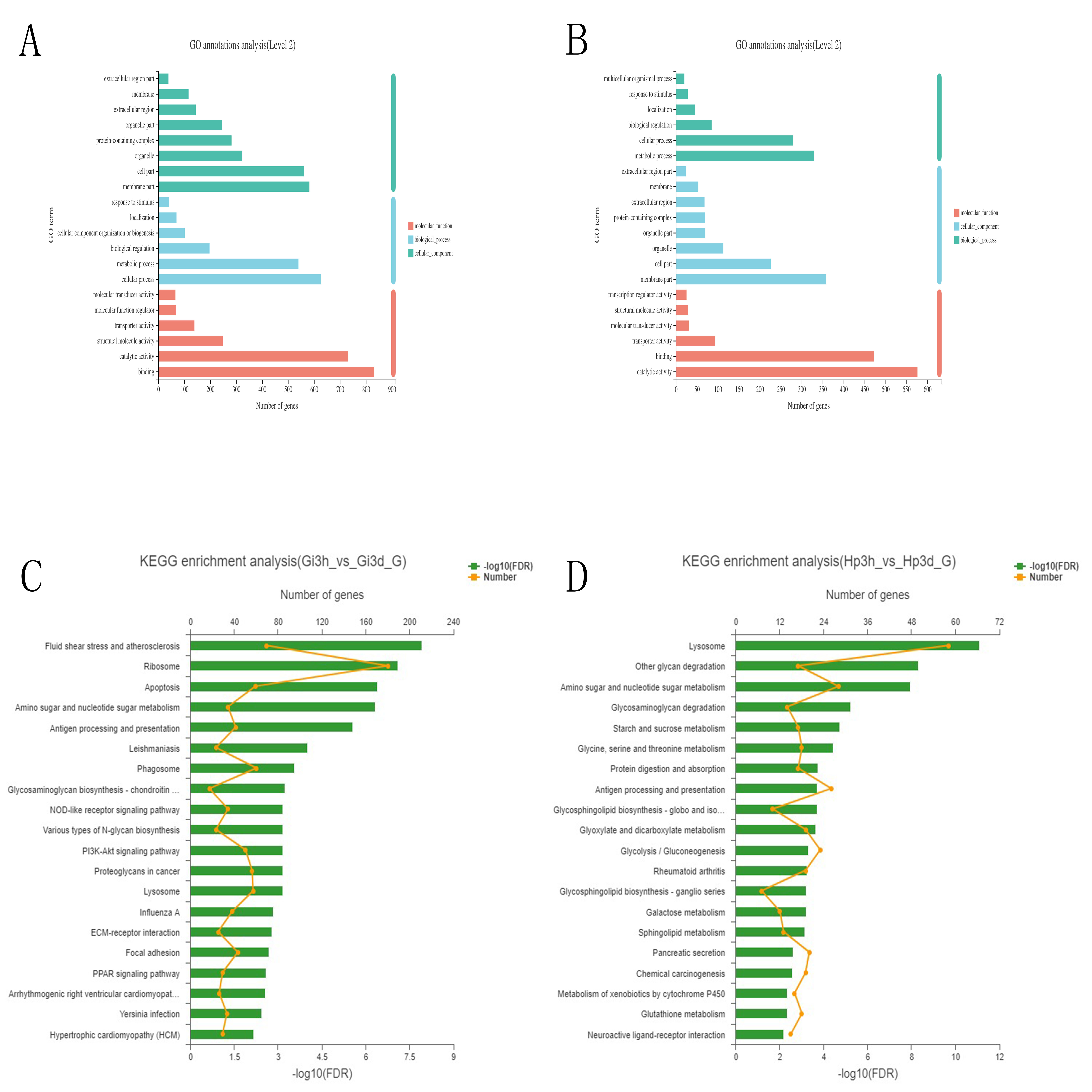

Supplement: Supplementary file 2 — Supplementary Information 2. [file 41598_2021_95709_MOESM2_ESM.jpg]
